# Feasibility and validity of telephone triage for adverse events during a voluntary medical male circumcision campaign in Swaziland

**DOI:** 10.1186/1471-2458-14-858

**Published:** 2014-08-18

**Authors:** Tigistu Adamu Ashengo, Jonathan Grund, Masitsela Mhlanga, Thabo Hlophe, Munamato Mirira, Naomi Bock, Emmanuel Njeuhmeli, Kelly Curran, Elizabeth Mallas, Laura Fitzgerald, Rhoy Shoshore, Khumbulani Moyo, George Bicego

**Affiliations:** Maternal and Child Health Integrated Program (MCHIP), and Jhpiego—an affiliate of the Johns Hopkins University, Washington, DC USA; Division of Global HIV/AIDS, Center for Global Health, Centers for Disease Control and Prevention (CDC), Atlanta, GA USA; Ministry of Health, Lobamba, Mbabane, Kingdom of Swaziland; United States Agency for International Development, Lobamba, Mbabane, Kingdom of Swaziland; United States Agency for International Development, Washington, DC USA; Futures Group, Lobamba, Mbabane, Kingdom of Swaziland; Family Life Association of Swaziland, Lobamba, Mbabane, Kingdom of Swaziland; Population Services International, Lobamba, Mbabane, Kingdom of Swaziland; Department of International Health, Johns Hopkins Bloomberg School of Public Health, Baltimore, MD USA

## Abstract

**Background:**

Voluntary medical male circumcision (VMMC) reduces HIV acquisition among heterosexual men by approximately 60%. VMMC is a surgical procedure and some adverse events (AEs) are expected. Swaziland’s Ministry of Health established a toll-free hotline to provide general information about VMMC and to manage post-operative clinical AEs through telephone triage.

**Methods:**

We retrospectively analyzed a dataset of telephone calls logged by the VMMC hotline during a VMMC campaign. The objectives were to determine reasons clients called the VMMC hotline and to ascertain the accuracy of telephone-based triage for VMMC AEs. We then analyzed VMMC service delivery data that included date of surgery, AE type and severity, as diagnosed by a VMMC clinician as part of routine post-operative follow-up. Both datasets were de-identified and did not contain any personal identifiers. Proportions of AEs were calculated from the call data and from VMMC service delivery data recorded by health facilities. Sensitivity analyses were performed to assess the accuracy of phone-based triage compared to clinically confirmed AEs.

**Results:**

A total of 17,059 calls were registered by the triage nurses from April to December 2011. Calls requesting VMMC education and counseling totaled 12,492 (73.2%) and were most common. Triage nurses diagnosed 384 clients with 420 (2.5%) AEs. According to the predefined clinical algorithms, all moderate and severe AEs (153) diagnosed through telephone-triage were referred for clinical management at a health facility. Clinicians at the VMMC sites diagnosed 341 (4.1%) total clients as having a mild (46.0%), moderate (47.8%), or severe (6.2%) AE. Eighty-nine (26%) of the 341 clients who were diagnosed with AEs by clinicians at a VMMC site had initially called the VMMC hotline. The telephone-based triage system had a sensitivity of 69%, a positive predictive value of 83%, and a negative predictive value of 48% for screening moderate or severe AEs of all the AEs.

**Conclusions:**

The use of a telephone-based triage system may be an appropriate first step to identify life-threatening and urgent complications following VMMC surgery.

## Background

More than 40 observational studies and three randomized controlled trials have demonstrated that voluntary medical male circumcision (VMMC) reduces HIV acquisition among heterosexual men by approximately 60% [1–4]. Extended follow-up at 66 months (Kenya) and 5 years (Uganda) also exhibited sustained reductions in HIV incidence of 64% and 73% respectively [5, 6]. In Orange Farm, South Africa, a cross-sectional study of HIV incidence found that HIV incidence was reduced by 76% in circumcised versus [7] confirming the long term prevention benefits of male circumcision. Given the potential impact of VMMC on HIV incidence, the World Health Organization (WHO) and the Joint United Nations Programme on HIV/AIDS (UNAIDS) recommended that VMMC should be part of comprehensive HIV prevention programming in regions with generalized HIV epidemics and low levels of male circumcision [8]. Modeling projections suggest that approximately 3.36 million new HIV infections and 386,000 AIDS deaths would be averted through 2025 if 80% coverage with VMMC in 14 priority countries in Eastern and Southern Africa were achieved within five years [9]. In order to achieve this level of coverage, an estimated 20 million VMMC procedures would need to be performed [9].

Male circumcision is a minor surgical procedure that involves risk. Some adverse events (AEs) are expected, even when surgery is conducted in sanitary settings by trained clinicians. Several factors can minimize complications following VMMC, including client assessment and history-taking; screening for sexually transmitted infections (STIs), genital anomalies, and bleeding disorders; use of skilled VMMC providers; correct post-operative client instruction and care; client adherence to post-operative instructions; and appropriate monitoring, assessment, and management of AEs. All complications must be closely monitored to ensure that clients receive appropriate post-operative clinical management. Timely identification, proper triage and management, and accurate reporting of AEs following circumcision are critical to maintaining high-quality, safe VMMC service delivery.

AE classifications are generally defined as intra-operative if they occur during surgery or before discharge from the clinic, or post-operative if they occur following discharge from the clinic. AEs are also classified by severity (mild, moderate, or severe). Mild AEs are generally understood to be part of routine surgery and are easily managed (e.g., post-operative bleeding that is easily controlled with pressure, swelling that resolves over time). Moderate and severe AEs may require further treatment, such as antibiotics, or re-exploration of the surgical site. Definitions of AE types associated with VMMC vary slightly by country but generally include bleeding, infection, pain, swelling (hematoma), reaction to local anesthesia, wound disruption, damage to penis, sexual or erectile dysfunction, scarring, torsion of penis, excessive or insufficient skin removed, and difficulty urinating. Both intra- and post-operative AEs related to VMMC surgery are uncommon [10–12].

Data on AEs following elective adolescent and adult circumcision are limited. In the three randomized controlled trials on VMMC (in Kenya, South Africa, and Uganda), the mean combined rate for all AEs was 5.0%, although adherence to post-operative instructions for wound care may have been better in trial settings than in routine service delivery [1–3, 13, 14]. The trials also used different AE definitions, procedures for active follow-up surveillance, and calculations for AEs, which could account for the range in the overall AE rate: 1.7%, 3.8%, and 7.6% in the Kenyan, South African, and Ugandan clinical trials, respectively [1–3]. Programmatic evidence from adolescent and adult VMMC programs suggests that AEs are generally uncommon, with post-operative infection and bleeding reported most frequently. A study of post-operative AEs in rural Kenya found that the prevalence of AEs was 1.3% among those returning for post-operative review within seven days of circumcision [15]. Different VMMC surgical techniques can be performed by doctors and non-physicians while maintaining low AE rates [16, 17]. During accelerated service delivery in Iringa, Tanzania, in 2010, the overall AE rate was less than 1%, and 80% of the AEs were categorized as mild or moderate, and only 20% were severe [18]. Similarly, the moderate and severe AE rate from Kenya’s 2010 Rapid Results Initiative, which provided VMMCs to more than 55,000 adolescent and adult males in 30 working days, was 1.5% among those adhering to post-operative follow-up recommendations [19]. In a study in Orange Farm, South Africa, which investigated the feasibility of a comprehensive VMMC program, more than 14,000 VMMC procedures were performed with a combined intra- and post-operative AE rate of 1.8%. [20].

Swaziland has the highest HIV prevalence in the world, at 31%, and prevalence is highest among females aged 15–25 years and males aged 25–33 years [21]. The HIV epidemic is largely driven by heterosexual transmission, and intergenerational sexual partnerships are common [22]. Swaziland’s Ministry of Health published a five-year male circumcision strategy in 2008 that highlighted the need for widespread VMMC scale-up in Swaziland [23]. Swaziland’s VMMC program was doctor-led with task-sharing among lower cadres, and service outlets were primarily based in public hospitals and stand-alone clinics. The minimum VMMC package was offered to all clients and included HIV testing and counseling, active exclusion of STIs, provision and promotion of condoms, risk reduction counseling, and circumcision surgery. All VMMC clients were advised to return for post-operative review 48 hours and seven days following surgery. Clients with post-operative AEs were assessed and treated according to the Ministry of Health approved AE guidelines. All clinicians working on VMMC were trained in the diagnosis and clinical management of AEs. All VMMC service delivery sites, including both fixed and temporary sites, were supplied with the necessary equipment, supplies, and medicines to treat AEs. Clients testing HIV-positive were referred to HIV care and treatment services for CD-4 testing, clinical staging, and antiretroviral treatment, as needed. All clients, regardless of HIV status, were offered VMMC, although HIV-infected clients were counseled on its limited benefits.

In hopes of controlling its HIV epidemic and reducing new infections, Swaziland planned an ambitious national VMMC campaign to be conducted in 2011 that would condense its five-year strategy into a single year. The campaign was called *Soka Uncobe* ("circumcise and conquer" in siSwati), and it aimed to provide VMMC surgery to approximately 150,000 adolescent and adult males aged 15–49 years. The campaign used the country’s existing VMMC sites and established several temporary sites to expand service delivery sites throughout the country.

Given the expected surge of VMMC clients during the campaign, and the corresponding increase in the number of AEs, Swaziland’s Ministry of Health implemented a telephone-based VMMC hotline. The toll-free hotline served two purposes: to give Swazi citizens the opportunity to ask trained nurses general questions about VMMC, and to triage post-operative AEs. The hotline utilized staff nurses from Swaziland’s Emergency Preparedness and Response (EPR) department who routinely handled a variety of disasters and emergencies by telephone.

In the VMMC clinics, all clients were given an appointment card that contained the date and location of their next follow-up visit. This card also contained the VMMC hotline number that they could call if they needed information, advice, or assistance regarding post-operative healing and management of any AEs. The hotline number was also included on advertisements and posters and during radio spots promoting the VMMC campaign throughout the country. Prospective clients and others interested in VMMC were recommended to call the hotline with questions about VMMC. The objectives of this analysis were to describe the reasons VMMC clients called the VMMC hotline and to assess the accuracy of telephone-based triage for VMMC in differentiating among mild, moderate, and severe AEs.

## Methods

### Description of telephone triage

**Figure 1:**
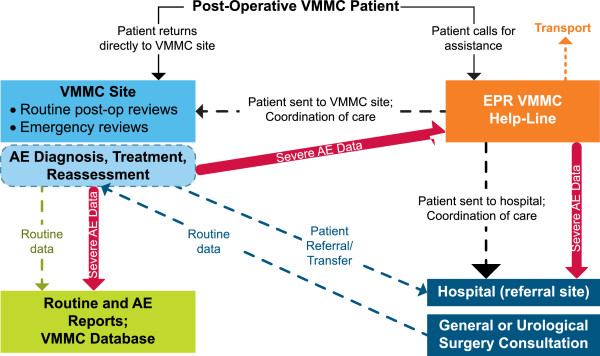
**Adverse event notification and management network.**

The nurses who provided telephone triage for VMMC completed a full course of competency-based clinical training on male circumcision under local anesthesia, which is the same training required of surgical nurses in VMMC facilities. Triage nurses were trained to assess VMMC clients’ concerns without the advantage of visual inspection or face-to-face interaction, by using comprehensive algorithms of AEs developed by a team of experienced VMMC-provider clinicians in Swaziland. They were required to rely on their communication skills and knowledge of the VMMC procedure, routine post-operative healing stages, and common post-operative AEs in order to accurately ascertain the client’s condition and needs. The function of the telephone triage nurse was to determine whether the caller had a VMMC-related complaint or sought information about VMMC. For those seeking information, the triage nurses provided general VMMC-related information and education VMMC service delivery in Swaziland. For those calling with suspected AEs, the triage nurses would use a series of algorithms to determine the cause and severity of the complaint. The triage nurse would then direct the caller to the appropriate emergency services, if necessary, and/or recommend medical follow-up based on established triage protocols (Figure 1). During the *Soka Uncobe* campaign, the EPR was staffed with five trained nurses who provided 24-hour coverage on a rotating basis.

**Figure 2:**
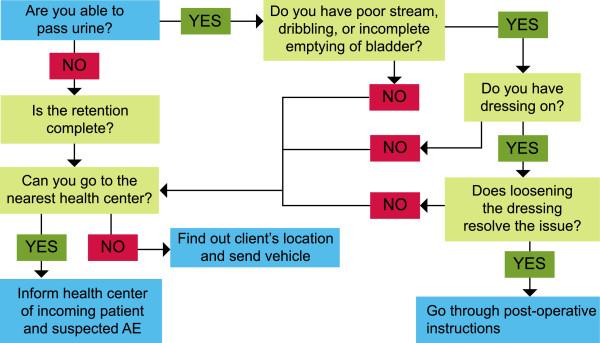
**Triage algorithm #1: Voiding difficulties: Nurse algorithm: Post-operative voiding difficulties (Inability to Pass Urine).**

**Figure 3:**
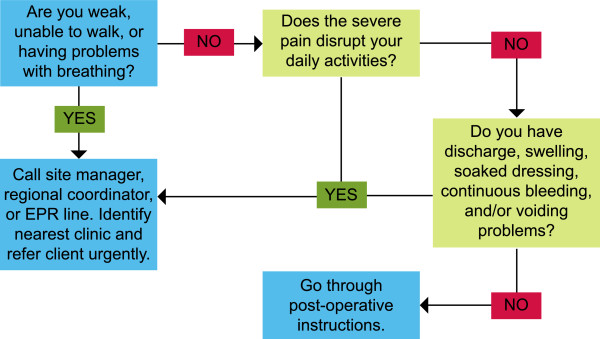
**Triage algorithm #2: Pain.**

**Figure 4:**
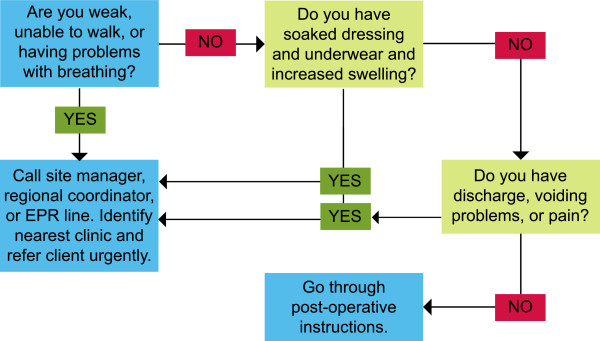
**Triage algorithm #3: Bleeding.**

**Figure 5:**
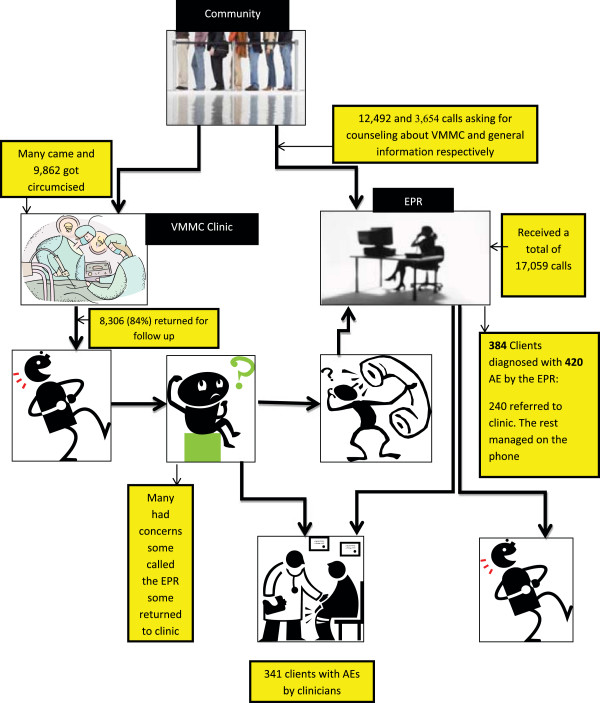
**Schematic representation of relationship between community, VMMC clinic, and the EPR (Phone Triage Center).**

Treatment algorithms were created for four common AEs that have the potential to become severe: bleeding, voiding difficulty, pain, and infection. Triage nurses utilized the algorithms to guide their telephone-based clinical decision-making (Figures 2, 3 and 4). Nurses assessed a caller’s symptoms first by determining the most acute and urgent conditions in order to determine the need for emergency intervention. Depending on the caller’s responses, the nurses followed specific procedures to obtain the necessary information and determine a course of action, as per the clinical recommendations [11]. As soon as triage nurses ruled out the possibility of critical or life-threatening AEs, they determined the type and severity of the complaint. If a nurse determined the complaint to be less serious than a mild AE, the nurse provided reassurance and information on wound care. If a nurse determined that a caller was experiencing a moderate or severe AE, he/she directed the caller to the nearest health facility for treatment. If the caller needed emergency transport to the clinic, then the nurse arranged for a vehicle to be sent to the caller’s location, free of charge. The nurse also informed the receiving site of the client’s condition and pending arrival (Figure 5).

We retrospectively reviewed a dataset of telephone calls logged by the triage nurses during the *Soka Uncobe* VMMC campaign. The call data included purpose of the call, AE type and severity for those experiencing complications, and algorithm used for telephone triage. We analyzed service delivery data at VMMC sites/clinics that included VMMC date and AE type and severity, as confirmed by a VMMC clinician. Both datasets were de-identified and did not contain any personal identifiers. Proportions of AEs were calculated from the VMMC hotline call data and from service delivery data recorded by the VMMC health facilities. Sensitivity and specificity analyses were performed to assess the validity of phone-based triage compared to clinically confirmed moderate and severe AEs for those clients with one or more AEs and were assessed by both the triage nurses and the clinicians at VMMC clinics.

A non-research determination and approval for secondary data analysis was provided by the Ministry of Health of the Kingdom of Swaziland and U.S. Centers for Disease Control and Prevention IRB for the scale-up project entitled: "*Design and implementation of a complete set of tools and procedures to monitor and evaluate a national scale up of MC for Swaziland"* 2011-2021.

## Results

### Calls to the VMMC hotline and adverse events identified through telephone triage

At the end of *Soka Uncobe* campaign, call data were gathered from April 13, 2011, to December 29, 2011. A total of 17,059 calls were registered by the triage nurses. Calls requesting VMMC education and counseling were most common, totaling 12,492 (73.2%). Five hundred (2.9%) of the calls received were complaints that the triage nurses managed by phone (Table 1). The triage nurses diagnosed 384 clients with one or more AEs and 420 (2.5%) total AEs, according to the algorithms. Of these, 269 (63.8%) callers were diagnosed with a mild AE, 139 (33.1%) with a moderate AE, and 12 (3.1%) with a severe AE (Table 2). According to the predefined clinical algorithms, all moderate and severe AEs (153) diagnosed by the triage nurses were referred for clinical management at a health facility. An additional 87 calls did not meet the moderate or severe definitions but were self referred or referred by the triage nurses to reassure the clients. Thus, a total of 240 calls were referred. The most common AEs diagnosed through telephone triage were bleeding (28.6%), infection (27.6%), swelling (24.8%), pain (8.6%), and wound disruption (6.9%) (Table 3). Total call volume was highest in the middle of the campaign—week 43—and VMMC hotline averaged 437 calls per week.

**Table 1:** **Distribution of calls to the VMMC hotline (n = 17,059)**

EPR call distribution	Frequency
VMMC education and counseling	12,492 (73.2%)
Information about VMMC site locations and transport	3,654 (21.4%)
General post-operative complaints-reassured by phone	500 (2.9%)
Referred/site-managed adverse events	240 (1.4%)
Other calls	173 (1.0%)
Total	17,059 (100%)

**Table 2:** **Severity of AEs diagnosed by telephone triage**

Severity of AEs diagnosed by the EPR	Frequency
Mild	269 (64.0%)
Moderate	139 (33.1%)
Severe	12 (2.9%)
Total	420 (100%)

**Table 3:** **Types of AEs diagnosed by telephone triage**

Adverse event	Frequency	Percentage
Bleeding	120	28.6%
Infection	116	27.6%
Swelling, including Hematoma	104	24.8%
Pain	36	8.6%
Wound disruption	29	6.9%
Voiding difficulty	10	2.4%
Other	5	1.2%
Total	420	(100%

### Adverse events identified in the VMMC service delivery sites

During the *Soka Uncobe* campaign, from March through December 2011, 9,862 VMMCs were performed at 29 clinics/sites. Of the 9,862 clients undergoing VMMC surgery, 8,306 (84.2%) returned for routine post-operative care within seven days, as recommended. A total of 341 clients had a mild, moderate, or severe AE (341/8,306, or 4.1%). The most common AEs were infection (184), swelling (44), and bleeding (39). Most AEs were mild (157, or 46.0%) or moderate (163, or 47.8%), while severe AEs were least frequent (21, or 6.2%), resulting in a post-operative moderate and severe AE rate of 2.2% (184/8,306) among those who returned for follow-up at a VMMC site during the campaign (Table 4). All AEs were clinically managed by the VMMC staff.

**Table 4:** **Severity and types of AEs diagnosed through clinical examination at the VMMC Sites during** ***Soka Uncobe*** **(N = 341)**

	Severity		
Adverse events (N = 431)	Mild (n = 157; 46%)	Moderate (n = 163; 47.8%)	Severe (n = 21; 6.2%)
Infection	82 (52.2%)	96 (58.9%)	6 (28.6%)
Swelling	25 (15.9%)	21 (12.9%)	5 (23.8%)
Wound disruption/dehiscence	20 (12.7%)	18 (11.0%)	4 (19.1%)
Bleeding	14 (8.9%)	14 (8.6%)	3 (14.3%)
Pain	11 (7.0%)	NA	NA
Others	5 (3.2%)	14 (8.6%)	3 (14.3%)

### Adverse events screened by telephone triage and confirmed by clinical diagnosis

Clinicians at the VMMC sites diagnosed 341 clients with mild, moderate, or severe AEs from April to December 2011. Of these, 89 (48.4%) had initially called the VMMC hotline with complaints following their VMMC. Fifty-two (58.4%) were referred by the triage nurses to a VMMC site as the telephone triage indicated that they had a moderate or severe AE. The triage nurses determined that 37clients (41.6%) did not have a moderate or severe AE according to the algorithms, but these clients went to clinic without referral or were referred to reassure them. Of the 52 clients who called the VMMC hotline and were diagnosed with a moderate or severe AE through telephone triage, 43 clients (82.7%) were confirmed to have a moderate or severe AE by VMMC site clinicians, and 9 clients (17.3%) were clinically diagnosed to not have a moderate or severe AE. The AE algorithms used by the triage nurses therefore had a sensitivity of 69%, a positive predictive value of 83%, and a negative predictive value of 48% (Table 5) in identifying moderate and severe adverse events from mild adverse events.

**Table 5:** **Validity of AE algorithms by clinical confirmation of triage nurse diagnoses (n = 89)**

Characteristic	Diagnosed as moderate or severe AE by VMMC site clinicians	Diagnosed as not moderate or severe AE by VMMC site clinicians	Total	
Diagnosed as moderate or severe AE by telephone triage	***43***	***9***	52	Positive predictive value = 82.7%
Diagnosed as not moderate or severe AE by telephone triage	***19***	***18***	37	Negative predictive value = 48.6%
Total	62	27	89	
	Sensitivity = 69.4%	Specificity = 66.7%		

Note: Sensitivity = a/(a + c); specificity = d/(b + d); positive predictive value = a/(a + b); negative predictive value = d/(c + d).

## Discussion

The use of a telephone triage system within an EPR department can be an appropriate first step in not only identifying life-threatening and urgent complications following VMMC surgery but also in providing information to community members with concerns and questions about VMMC services in Swaziland. A substantial proportion of hotline callers sought general information and education about VMMC, which offered insights into community perceptions of the intervention. These insights can help in crafting more precise messages for more effective VMMC communication. They also indicate a need for continuous community-level dialogue about the role of biomedical interventions in mitigating HIV transmission in Swaziland.

This analysis focused on documenting the reasons why VMMC clients called the hotline and the validity of the AE triage through telephone calls. WHO/UNAIDS recommends that VMMC clients return to the circumcising facility within seven days of surgery for routine post-operative review [11]. The use of the VMMC hotline provided clients with an additional opportunity to communicate with trained clinicians about the healing of their wound and about VMMC in general. Several VMMC programs and studies have investigated the use of telephone or text-based reminders about follow-up visits and post-operative sexual abstinence [24–27]. To our knowledge, this is the first evaluation of an AE triage system that has been employed for notification and management of AEs during a VMMC campaign.

The AE triage system used during Swaziland’s *Soka Uncobe* campaign demonstrated that trained nurses using clinical algorithms can provide effective reassurance and simple management for AEs deemed mild and provide referrals for moderate and severe post-operative AEs that are later confirmed and managed by clinicians at clinics. Many questions remain about the effectiveness of telephone-based triage for VMMC. It is possible that telephone-based triage would work most effectively to provide further reassurance to post-operative clients with mild complaints and save time and cost of travel to clinic. It may also function as a complement to the in-person, post-operative review process, rather than as a replacement for it.

VMMC provides a unique opportunity to provide health services to adolescent and adult males with otherwise limited access to such services. The hotline is a relatively simple mechanism that has the potential to introduce a large audience to the country’s health services. Beyond providing referrals to health clinics, the telephone-based triage can also provide general information about the benefits of VMMC, locations of fixed and mobile service delivery sites, and other basic questions related to men’s health and encourage utilization of services.

While campaigns are an effective format to deliver VMMC for HIV prevention, they may add further stress to a delicate health care system. VMMC campaigns in other countries have emphasized the required human resource and logistics needs [18, 19, 28, 29], and having nurses staff a VMMC hotline would add to this burden. However, a telephone-based system may help reduce the number of clients visiting health facilities who do not otherwise need to see a clinic-based service provider for their post-operative questions or concerns. VMMC clients with suspected mild AEs can be given clinical instructions to manage their wounds. In many cases, this would eliminate the need for the client to return to the VMMC facility for additional follow-up care. In addition, the use of a simplified diagnostic tool, such as a clinical algorithm, could offer an opportunity to implement task-shifting. Relying on clinical algorithms to diagnose and refer the most severe complications may alleviate the burden on high-level health care workers, who are in short supply in the Eastern and Southern African countries where VMMC is being scaled up.

There are several limitations in this analysis. Initial documentation was incomplete for clients’ subsequent outcomes after AE-related calls to the hotline. It is also possible that some clients might have accessed services in non-*Soka Uncobe* clinics, including hospitals not participating in the campaign or private clinics, so these clients were not tracked. While almost three-quarters of households in Swaziland have access to a telephone [30], it is possible that some people were unable to access the hotline. In addition, the periods of analysis for the VMMC hotline and VMMC service delivery sites varied, which may have resulted in underreporting of calls made to the hotline between March and April 2011.

## Conclusion

The experience in Swaziland shows that the use of a telephone-based triage system may be an appropriate first step to identify life-threatening and urgent complications following VMMC surgery. This is especially essential since safety as well as community perception of safety in large-scale surgical services is a critical component of acceptability. While this review demonstrates the potential utility of this type of notification and management system, the costs of establishing the system have not yet been established. An important next step will be to determine the cost of the system in its initial and recurrent elements and thus its viability and sustainability in Swaziland and other countries where VMMC scale-up is part of the national HIV prevention strategy.

### Ethical approval

A non-research determination status was obtained for all routinely collected VMMC data in Swaziland through the scientific unit of the Centers for Disease Control and Prevention (The NRD is attached as a separate file).

## Abbreviations

AE: Adverse event
EPR: Emergency Preparedness and Response
PEPFAR: President’s Emergency Plan for AIDS Relief
STI: Sexually transmitted infection
UNAIDS: Joint United Nations Programme on HIV/AIDS
VMMC: Voluntary medical male circumcision
WHO: World Health Organization.
